# Physiological role of the 3′IgH CBEs super-anchor in antibody class switching

**DOI:** 10.1073/pnas.2024392118

**Published:** 2021-01-13

**Authors:** Xuefei Zhang, Hye Suk Yoon, Aimee M. Chapdelaine-Williams, Nia Kyritsis, Frederick W. Alt

**Affiliations:** ^a^Howard Hughes Medical Institute, Boston Children's Hospital, Boston, MA 02115;; ^b^Program in Cellular and Molecular Medicine, Boston Children’s Hospital, Boston, MA 02115;; ^c^Department of Genetics, Harvard Medical School, Boston, MA 02115

**Keywords:** class switch recombination, chromatin loop extrusion, promoter competition, 3'IgH CBEs

## Abstract

IgH class switch recombination (CSR) replaces Cμ constant region (C_H_) exons with one of six downstream C_H_s by joining transcription-targeted double-strand breaks (DSBs) in the Cμ switch (S) region to DSBs in a downstream S region. Chromatin loop extrusion underlies fundamental CSR mechanisms including 3′IgH regulatory region (3′IgHRR)-mediated S region transcription, CSR center formation, and deletional CSR joining. There are 10 consecutive CTCF-binding elements (CBEs) downstream of the 3′IgHRR, termed the “3′IgH CBEs.” Prior studies showed that deletion of eight 3′IgH CBEs did not detectably affect CSR. Here, we report that deletion of all 3′IgH CBEs impacts, to varying degrees, germline transcription and CSR of upstream S regions, except that of Sγ1. Moreover, deletion of all 3′IgH CBEs rendered the 6-kb region just downstream highly transcribed and caused sequences within to be aligned with Sμ, broken, and joined to form aberrant CSR rearrangements. These findings implicate the 3′IgH CBEs as critical insulators for focusing loop extrusion-mediated 3′IgHRR transcriptional and CSR activities on upstream C_H_ locus targets.

## Significance

B lymphocytes change antibody heavy chain (IgH) isotypes by a recombination/deletion process called IgH class switch recombination (CSR). CSR involves introduction of DNA breaks into a donor switch (S) region and also into one of six downstream S regions, with joining of the breaks changing antibody isotype. A chromatin super-anchor, of unknown function, is located just downstream of the IgH locus. We show that complete deletion of this super-anchor variably decreases CSR to most S regions and creates an ectopic S region downstream of IgH locus that undergoes aberrant CSR-driven chromosomal rearrangements. Based on these and other findings, we conclude that the super-anchor downstream of IgH is a critical insulator for focusing potentially dangerous CSR rearrangements to the IgH locus.

Mature B cells undergo immunoglobulin (Ig) heavy chain (IgH) class switch recombination (CSR) to change the constant region of IgH chains and modulate antibody effector functions (1, 2). Transcription from IgH V(D)J exons runs through proximal Cμ exons that specify IgM antibodies. Upon activation, B cells undergo CSR to replace Cμ with one of six sets of constant region exons (C_H_s) to produce other antibody isotypes (IgG, IgE, or IgA) (2, 3). Both the intronic enhancer (iEμ), located at the 5′ end, and the 3′IgH regulatory region (3′IgHRR) superenhancer, located at the 3′ end, of the IgH constant region locus contribute to CSR (4–9). The 3′IgHRR plays critically important roles in CSR by interacting with I promoters upstream of targeted S regions to activate C_H_ transcription in a cytokine and activation-specific manner (10, 11). Then, transcriptionally targeted activation-induced cytidine deaminase (AID) (12) initiates double-strand breaks (DSBs) in downstream acceptor S regions that can join in deletional orientation to AID-initiated DSBs in the donor S upstream of Cμ (Sμ) (1). Insertion of active promoters in various C_H_ locus sites inhibits I-promoter activation and CSR in upstream promoters (except Iγ1) but not in downstream promoters, suggesting that linear competition over 100-kb distances of I promoters for 3′IgHRR activation contributes to CSR regulation (13).

Regulated chromatin loop extrusion provides mechanistic underpinnings for the overall CSR mechanism by promoting synapsis of enhancers, promoters, S regions, and DSB ends necessary for productive, deletional CSR (11). In naive B cells, cohesin is loaded onto the chromatin around the IgH enhancers (iEμ or 3′IgHRR) and then extrudes the 3′IgHRR into proximity with the iEμ-Sμ region to form a dynamic CSR center (CSRC) containing donor Sμ and the two involved enhancer regions (11). In addition to loading cohesin, these enhancer regions appear to also function as dynamic loop extrusion impediments that contribute to formation of a CSRC (11). In CSR-activated B cells, loop extrusion also brings cytokine/activator-primed I promoters into the CSRC where they can be further transcriptionally activated by IgH enhancers, resulting in further cohesin loading, loop extrusion, and alignment with the transcribed acceptor S region with the donor Sμ region for S-S synapsis and AID targeting (11). For the next joining step, it has been implicated that cohesin rings put tension on the S regions synapsed in the CSRC to promote AID-initiated DSB ends in donor and acceptor S regions to be dominantly joined in deletional orientation for CSR. Based on this model, after AID initiates DSBs, one or both break-ends within an S region are reeled into an opposing cohesin ring where the extrusion process is stalled. Then a break in the other S region will have the same fate, eventually aligning the break-ends for deletional CSR joining (11). The model also has been proposed to explain how DSBs within ectopic S regions (non–S-region sequences) formed by CTCF-binding elements (CBEs) insertions into the C_H_ region also are synapsed with Sμ in the CSRC, after which their infrequent AID-initiated DSBs are joined in deletional orientation to AID-initiated Sμ DSBs (11).

The 10 consecutive CBEs downstream of 3′IgHRR, variously termed the 3′IgH CBEs or the 3′IgH locus superanchor, have been speculated to function as an insulator at 3′ end of the IgH locus (14–16). However, deletion of the first 8 of the 10 3′IgH CBEs showed little effect on CSR in mice (17). Deletion of all 10 3′IgH CBEs in CH12F3 cells revealed modest effects on the transcription and CSR to Cα (15). However, transcription of the 3′IgHRR and the Cα region appears to be constitutively activated in CH12F3 cells versus normal B cells, being much more robust and extending more than 20 kb downstream through the 3′IgH CBEs and beyond (11). Thus, CH12F3 cells do not necessarily provide an accurate model for studying potential roles of 3′IgH CBEs in physiological CSR. As complete deletion of the 3′IgH CBEs in normal B-cell CSR has not yet been assayed, it remains unknown whether or not the 3′IgH CBEs play any potential direct or indirect roles in the physiological CSR process. Here, we describe experiments in which all 10 3′IgH CBEs were deleted (“complete 3′IgH CBEs-deleted”) on both alleles in embryonic stem (ES) cells that were then used for RAG2-deficient blastocyst complementation (RDBC) (18) to generate chimeric mice in which all mature B cells harbor the complete 3′IgH CBEs deletion. Our current studies of CSR in complete 3′IgH CBEs-deleted B cells indeed revealed roles for the 3′IgH CBEs in physiological CSR and clear-cut function for 3′IgH CBEs as physiological CSR insulators.

## Results

### Complete 3′IgH CBEs Deletion Decreases CSR to Most S Regions.

**Fig. 1.**
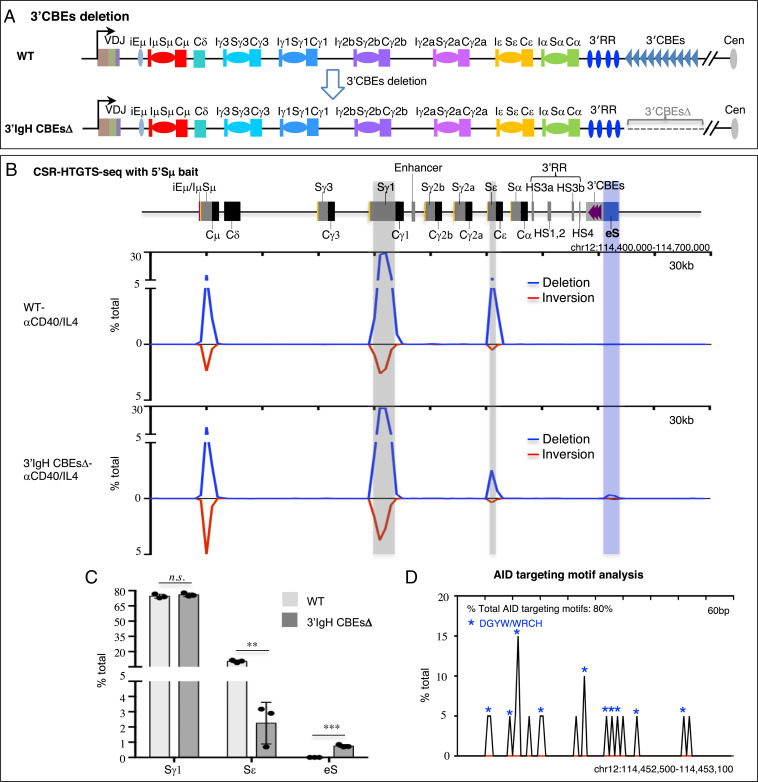
The 3′IgH CBEs deletion decreases Sε CSR and creates an eS region for aberrant translocation in αCD40/IL4-stimulated B cells. (*A*) Schematic of IgH locus from iEμ to 3′IgH CBEs and illustration of the generation of 3′IgH CBEs-deleted ES cells. (*B*) CSR-HTGTS-seq analysis of break joining between 5′Sμ and downstream acceptor S or non-S regions in WT and 3′IgH CBEs-deleted splenic B cells stimulated with αCD40/IL4. The blue line indicates deletional joining, and the red line indicates inversional joining. Gray bars highlight the Sγ1 and Sε. A blue bar highlights the ectopic S region (labeled as “eS”) just downstream of 3′IgH CBEs. (*C*) Bar graph showing percentages of junctions located in different S regions and the eS region from WT and 3′IgH CBEs-deleted splenic B cells stimulated with αCD40/IL4. Data represent mean ± SD from three independent repeats. *P* values were calculated via an unpaired two-tailed *t* test; *n.s.* indicates *P* > 0.05, **P ≤ 0.01, ***P ≤ 0.001. The raw data for this bar graph are summarized in *SI Appendix*, Table S1. (*D*) AID-targeting-motif analysis for the junctions located in a 600-bp region within the AID-targeted eS region from 3′IgH CBEs-deleted splenic B cells stimulated with αCD40/IL4. Blue asterisks indicate DGYW/WRCH motifs.

**Fig. 2.**
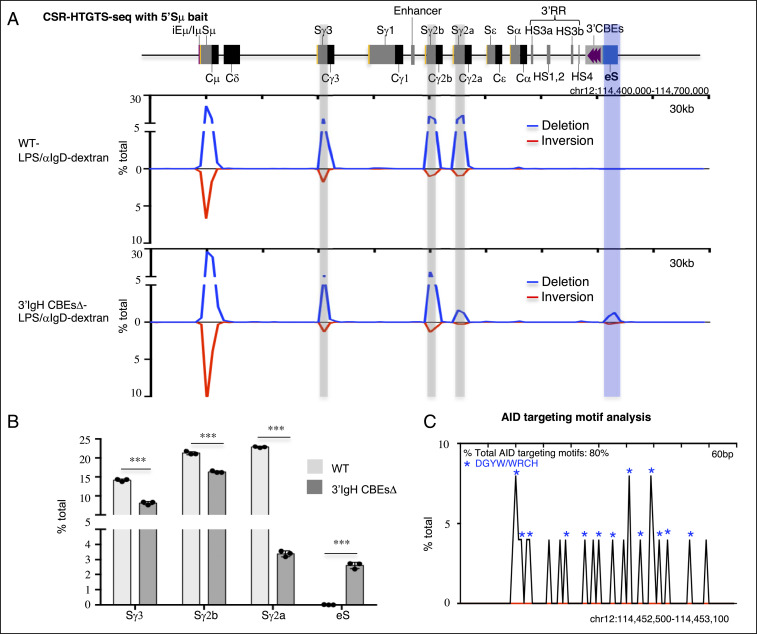
The 3′IgH CBEs deletion decreases Sγ3, Sγ2b, and Sγ2a CSR and creates an eS region for aberrant translocation in LPS/αIgD-dextran–stimulated B cells. (*A*) CSR-HTGTS-seq analysis of break joining between 5′Sμ and downstream acceptor S or non-S regions in WT and 3′IgH CBEs-deleted splenic B cells stimulated with LPS/αIgD-dextran. The blue line indicates deletional joining, and the red line indicates inversional joining. Gray bars highlight the Sγ1, Sγ2b, and Sγ2a. A blue bar highlights the ectopic S region (labeled as “eS”) just downstream of 3′IgH CBEs. (*B*) Bar graph showing percentages of junctions located in different regions from WT and 3′IgH CBEs-deleted splenic B cells stimulated with LPS/αIgD-dextran. Data represent mean ± SD from three independent repeats. *P* values were calculated via an unpaired two-tailed *t* test; ***P ≤ 0.001. The raw data for this bar graph are summarized in *SI Appendix*, Table S2. (*C*) AID-targeting-motif analysis for the junctions located in a 600-bp region within the AID-targeted eS region from 3′IgH CBEs-deleted splenic B cells stimulated with LPS/αIgD-dextran. Blue asterisks indicate DGYW/WRCH motifs.

While previous work indicated that deletion of 8 of the 10 CTCF-binding sites of the 3′IgH CBEs had little effect on class switching (17), it remains possible that the remaining two 3′IgH CBEs might mediate potential CSR functions. Therefore, to assess potential physiological roles of 3′IgH CBEs in CSR, we deleted all 10 of the 3′IgH CBEs in ES cells (Fig. 1*A* and *SI Appendix*, Fig. S1 *A* and *B*) and used our RDBC system (18) to generate chimeric mice in which all mature B cells derive from the donor 3′IgH CBEs-deleted ES cells. We isolated the primary splenic B cells from the wild-type (WT) and 3′IgH CBEs-deleted RDBC chimeras and stimulated the cells for 96 h with either αCD40/IL4 to induce class switching to Sγ1 and Sε or with LPS/αIgD-dextran to induce CSR to Sγ3, Sγ2b, and Sγ2a (19). Subsequently, we assayed for CSR by CSR-HTGTS-seq (11, 19). (*SI Appendix*, Fig. S1*C*). Approximately 75% of splenic B cells activated with αCD40/IL4 switched to Sγ1, and ∼10% switched to Sε (Fig. 1 *B* and *C* and *SI Appendix*, Fig. S1*D*). While complete 3′IgH CBEs deletion had no significant effect on CSR to Sγ1, it decreased CSR to Sε to about 30% of normal levels (from 10.4 to 2.8%) (Fig. 1 *B* and *C* and *SI Appendix*, Fig. S1*D* and Table S1). In LPS/αIgD-dextran–treated splenic B cells, the complete 3′IgH CBE deletion modestly decreased CSR to Sγ3 to about 60% of WT B-cell levels (from 14.1 to 8.1%) and CSR to Sγ2b to about 75% of WT B-cell levels (from 21.3 to 16.3%) (Fig. 2 *A* and *B* and *SI Appendix*, Fig. S1*E* and Table S2). However, CSR to Sγ2a substantially decreased to about 15% (from 22.9 to 3.4%) of WT B-cell levels (Fig. 2 *A* and *B* and *SI Appendix*, Fig. S1*E* and Table S2). IgG1 surface staining of 4-d αCD40/IL4-stimulated B cells revealed no effect on IgG1 expression by the 3′IgH CBEs deletion (*SI Appendix*, Fig. S1*F*), consistent with CSR-HTGTS-seq data. Moreover, IgG3 and IgG2b surface staining of LPS/αIgD-dextran–stimulated B cells revealed decreased IgG3- and IgG2b-positive B-cell frequencies upon 3′IgH CBEs deletion (*SI Appendix*, Fig. S1*F*) similar to findings of CSR-HTGTS-seq analyses.

### The Complete 3′IgH CBEs Deletion Decreases the Transcription of Most S Regions.

**Fig. 3.**
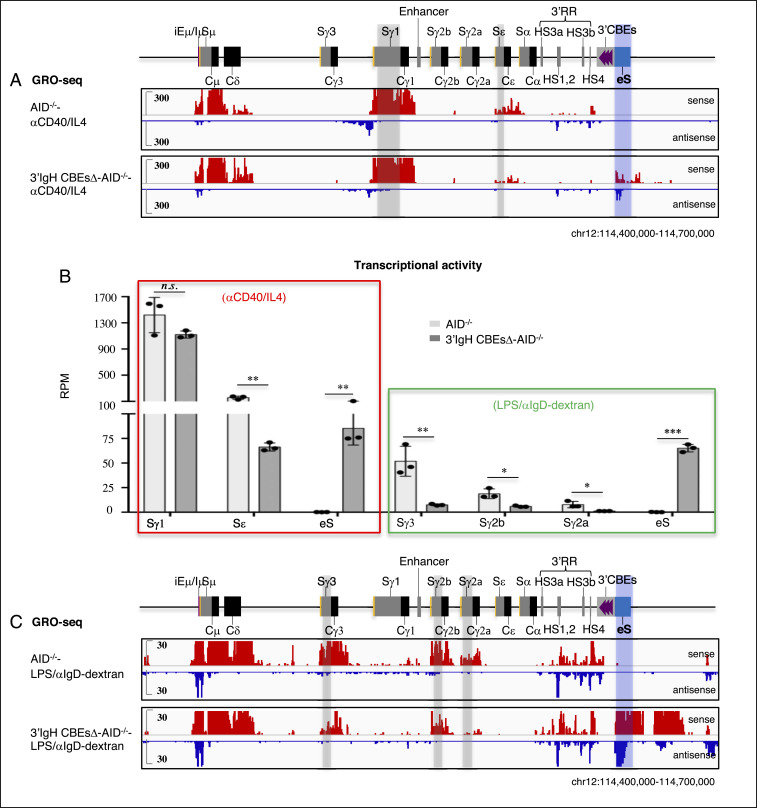
The 3′IgH CBEs deletion decreases transcription of the most upstream S region transcription and induces the transcription of the eS region. (*A*) GRO-seq profiles of the IgH locus from AID-deficient WT and 3′IgH CBEs-deleted splenic B cells stimulated with αCD40/IL4. Sense transcription is shown above in red, and antisense transcription is shown below in blue lines. Gray bars highlight the Sγ1 and Sε. A blue bar highlights the ectopic S region (labeled as “eS”) just downstream of the 3′IgH CBEs. (*B*) Bar graph shows GRO-seq transcriptional activity (calculated as RPM) of the different indicated S regions and the eS region in αCD40/IL4-or LPS/αIgD-dextran–stimulated AID-deficient WT and 3′IgH CBEs-deleted splenic B cells. Data represent mean ± SD from three independent repeats. *P* values were calculated via unpaired two-tailed *t* test; *n.s.* indicates *P* > 0.05, *P ≤ 0.05, **P ≤ 0.01, ***P ≤ 0.001. The raw data for this bar graph are summarized in *SI Appendix*, Tables S1 and S2. (*C*) GRO-seq profiles of the IgH locus from AID-deficient WT and 3′IgH CBEs-deleted splenic B cells stimulated with LPS/αIgD-dextran. Sense transcription is shown above in red, and antisense transcription is shown below in blue lines. Gray bars highlight the Sγ1, Sγ2b, and Sγ2a. A blue bar highlights the ectopic S region (labeled as “eS”) just downstream of the 3′IgH CBEs.

CSR is targeted to particular downstream acceptor S regions by activation/cytokine-induced transcription through them from an I-region promoter that lies just upstream of each of them. Induction of transcription from all I-region promoters, except that of Iγ1 (8, 13, 20, 21), is dependent on interactions with the 3′IgHRR enhancers. We used Global Run-on Sequencing (GRO-seq) to assess the transcription across the C_H_-containing portion of the IgH locus and immediately downstream sequences in WT and 3′IgH CBEs-deleted splenic B cells with or without CSR activation for 96 h. To obviate effects of CSR events on transcription patterns, we also deleted AID in WT and 3′IgH CBEs-deleted ES cells before use for RDBC. Treatment with αCD40/IL4, as expected (11, 19, 22), induced WT B cells to transcribe across Iγ1-Sγ1 and Iε-Sε (Fig. 3*A* and *SI Appendix*, Fig. S2). In approximate correspondence to CSR effects, the 3′IgH CBEs deletion had no significant effects on Iγ1-Sγ1 transcription but reduced Iε-Sε transcription to about 40% of WT B-cell levels (Fig. 3 *A* and *B* and *SI Appendix*, Fig. S2 and Table S1). Activation of splenic B cells with LPS/αIgD-dextran, as expected (19), induced transcription across Iγ3-Cγ3, Iγ2b-Cγ2b, and Iγ2a-Cγ2a (Fig. 3*C* and *SI Appendix*, Fig. S3). Compared to WT B-cell levels for this treatment, the 3′IgH CBEs deletion decreased Iγ3-Cγ3 transcription to about 15%, Iγ2b-Cγ2b transcription to about 30%, and Iγ2a-Cγ2a transcription to about 13% (Fig. 3 *B* and *C* and *SI Appendix*, Fig. S3 and Table S2). While the reduction in transcription levels across Iγ3-Cγ3, Iγ2b-Cγ2b, and Iγ2a-Cγ2a does not absolutely reflect the reduction in CSR levels to these C_H_ units in the LPS/aIgD-dextran–treated WT and 3′IgH CBEs-deleted B cells, general trends are similar. In this regard, we do not know the threshold of transcription for each S region required to promote given levels of CSR, which also are influenced by S-region sequence composition or length, among other potential factors (23, 24). Thus, we do not necessarily expect precise correspondence between CSR and transcription levels of a particular S region. These parameters have been compared in the context of mutations that affect CSR.

### The Complete 3′IgH CBEs Deletion Decreases S-Region Synapsis with the CSRC.

**Fig. 4.**
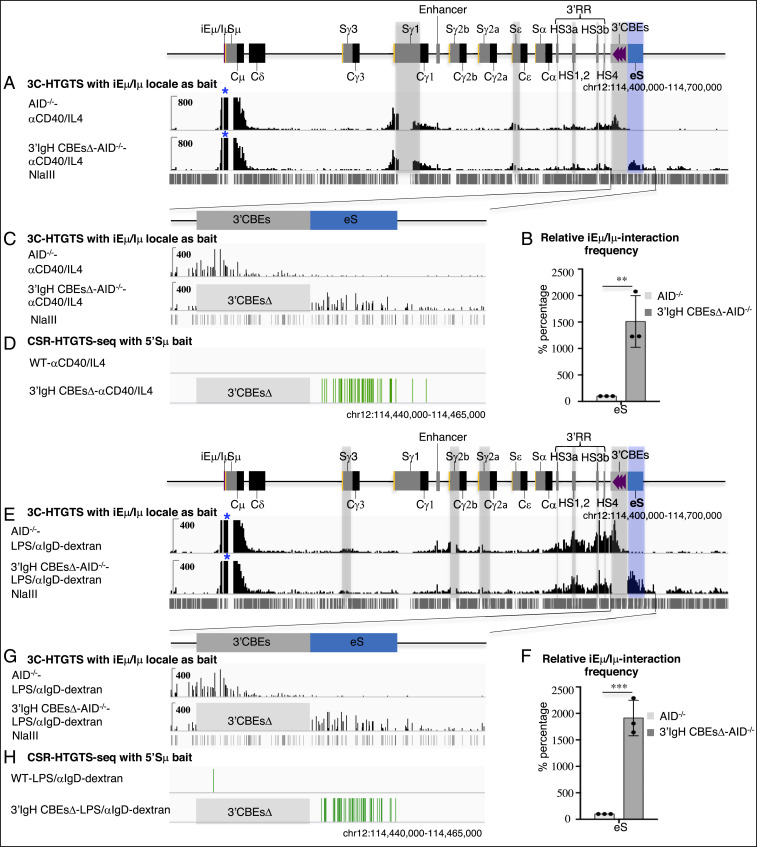
The 3′IgH CBEs deletion decreases most S-S synapsis and induces Sμ-eS synapsis for aberrant rearrangement. (*A*) 3C-HTGTS analysis of αCD40/IL4-stimulated AID-deficient WT and 3′IgH CBEs-deleted splenic B cells using iEμ/Iμ locale as bait (blue asterisk). Gray bars highlight the Sγ1, Sε, HS3a, HS1,2, HS3b, HS4 and 3′IgH CBEs. Blue bar highlight the ectopic S region (labeled as “eS”) just downstream of 3′IgH CBEs. (*B*) Bar graph shows the relative iEμ-Sμ interaction frequency with eS in αCD40/IL4-stimulated splenic B cells. Data represent mean ± SD from three independent repeats. *P* values were calculated via unpaired two-tailed *t* test; **P ≤ 0.01. The raw data for this bar graph are summarized in *SI Appendix*, Table S1. (*C*) Magnified 3C-HTGTS profiles in *A* to better reveal interaction patterns for the 3′IgH CBEs and eS region in αCD40/IL4-stimulated splenic B cells. (*D*) CSR-HTGTS-seq with 5′Sμ bait to show the rearrangement within the eS region from αCD40/IL4-stimulated WT and 3′IgH CBEs-deleted splenic B cells. (*E*) 3C-HTGTS analysis of LPS/αIgD-dextran–stimulated AID-deficient WT and 3′IgH CBEs-deleted splenic B cells using the iEμ/Iμ locale as bait (blue asterisk). Gray bars highlight the Sγ3, Sγ2b, Sγ2a, HS3a, HS1,2, HS3b, HS4, and 3′IgH CBEs. Blue bar highlights the ectopic S region (labeled as “eS”) just downstream of the 3′IgH CBEs. (*F*) Bar graph showing the relative iEμ-Sμ interaction frequency with eS in LPS/αIgD-dextran–stimulated splenic B cells. Data represent mean ± SD from three independent repeats. *P* values were calculated via unpaired two-tailed *t* test; ***P ≤ 0.001. The raw data for this bar graph are summarized in *SI Appendix*, Table S2. (*G*) Magnified 3C-HTGTS profiles in *E* to better reveal the interaction patterns for the 3′IgH CBEs and eS region in LPS/αIgD-dextran–stimulated splenic B cells. (*H*) CSR-HTGTS-seq with 5′Sμ bait to show the rearrangement within the eS region from LPS/αIgD-dextran–stimulated WT and 3′IgH CBEs-deleted splenic B cells.

Our prior studies, which employed the highly sensitive 3C-HTGTS chromatin interaction method (11), indicated that the transcribed iEμ-Sμ and the 3′IgHRR regions serve as dynamic loop extrusion impediments that can promote CSRC formation and S-S synapsis in CSRC to promote CSR. To assess potential effects of the complete 3′IgH CBEs deletion on S-region synapsis in the CSRC, we performed 3C-HTGTS with iEμ-Sμ bait in activated WT and 3′IgH CBEs-deleted splenic B cells. As noted previously, portions of Sμ and certain other S regions cannot be mapped by this assay due to lack of requisite NlaIII restriction endonuclease sites; thus, their interactions must be inferred from mappable sequences within them (11). In αCD40/IL4-stimulated B cells, the iEμ-Sμ locale significantly interacts with Sγ1 and Sε locales (Fig. 4*A* and *SI Appendix*, Fig. S4*A*). In this regard, the 3′IgH CBEs complete deletion had no significant effects on Sμ-Sγ1 synapsis while it modestly reduced Sμ-Sε synapsis to about 75% of WT B-cell levels (Fig. 4*A* and *SI Appendix*, Fig. S4*A* and Table S1). In LPS/αIgD-dextran–stimulated B cells, the iEμ-Sμ locale had relatively less interaction with Sγ3, Sγ2b, and Sγ2a (Fig. 4*E* and *SI Appendix*, Fig. S4*B*); however, the 3′IgH CBEs deletion significantly decreased Sμ-Sγ3 synapsis to about 45%, Sμ-Sγ3 synapsis to about 60%, and Sμ-Sγ3 synapsis to about 65% of WT B-cell levels (Fig. 4*E* and *SI Appendix*, Fig. S4*B* and Table S2). Again, the trend of these reductions is in the same direction as CSR, but also is subject, beyond mapping issues with some core S-region sequences, to the same comparison issues mentioned for correlation of S-region transcription levels for CSR levels above.

### 3′IgH CBEs Deletion Induces Transcriptional Activation and Abnormal Translocation of an Ectopic S Region to Compete with Upstream S Regions.

To further address the potential mechanism of the reduction of CSR to various S regions upon the complete deletion the 3′IgH CBEs, we employed GRO-seq to analyze transcription of sequences downstream of the 3′IgH CBEs in WT and complete 3′IgH CBEs-deleted nonstimulated splenic B cells. This analysis revealed that the 30-kb region just downstream of the 3′IgH CBEs was highly activated transcriptionally in nonstimulated, complete 3′IgH CBEs-deleted splenic B cells (*SI Appendix*, Fig. S5), suggesting that the 3′IgHRR may activate transcription of this downstream IgH region in the absence of the 3′IgH CBEs. Indeed, in contrast to the reduction transcription of various S regions (excluding Sγ1) in αCD40/IL4- or LPS/αIgD-dextran–stimulated splenic B cells, transcription of this immediately downstream IgH region was substantially increased in the absence of the 3′IgH CBEs (Fig. 3 *A*–*D* and *SI Appendix*, Figs. S2 and S3). Moreover, our 3C-HTGTS data showed that the iEμ-Sμ locale in the CSRC had greatly increased interactions with this transcriptionally activated region just downstream of the IgH locus upon deletion of the 3′IgH CBEs (*SI Appendix*, Fig. S4 *C*–*F*), indicating that, in the absence of the 3′IgH CBEs, this highly transcribed downstream region participates in substantial synapsis with Sμ within the CSRC (*SI Appendix*, Fig. S4 *A*, *B*, *D*, and *F*).

Transcription across the region downstream of IgH increased in both sense and antisense directions (Fig. 3 *A* and *C* and *SI Appendix*, Figs. S2 and S3), creating convergent transcription known to facilitate AID targeting (25). This region just downstream of the 3′IgH CBEs is juxtaposed to the 3′IgHRR enhancer after the complete 3′IgH CBEs deletion, likely leading to the transcriptional activation of the downstream region by the 3′IgHRR enhancer. The “sense” (defined by orientation sense transcription in the IgH locus) transcription is likely in large part due to continuation/extension of the 3′IgHRR transcription, while the antisense transcription which is initiated downstream of the 3′IgHRR may be activated at the ectopic promoter in this region promoted by the 3′IgHRR enhancer in the absence of the insulating 3′IgH CBEs.

Notably, αCD40/IL4 treatment of complete 3′IgH CBEs-deleted splenic B cells induced the aberrant translocations across the first 6 kb of this 30-kb transcribed sequence just downstream of the normal 3′IgH CBEs location, as indicated by junctions between Sμ and sequences across this region that accounted for nearly 1% of all CSR-related junctions (Fig. 1 *B* and *C* and *SI Appendix*, Fig. S1*D* and Table S1). In addition, LPS/αIgD-dextran treatment of complete 3′IgH CBEs-deleted splenic B cells also induced aberrant translocations between Sμ and sequences within this 6-kb sequence downstream of the normal 3′IgH CBEs location that accounted for nearly 3% of all CSR-related junctions (Fig. 2 *A* and *B* and *SI Appendix*, Fig. S1*E* and Table S2). Moreover, examination of junctions across a 600-bp “core” region of this 6-kb sequence downstream of the normal 3′IgH CBE location, which has the highest rearrangement frequency across this region, revealed that about 80% of the junctions within occurred within AID-targeting motifs under both aCD40/IL4 and LPS/aIgD-dextran stimulation conditions (Fig. 1*D* and Fig. 2*C*), consistent with their joining to Sμ via a CSR-related mechanism. The results are also striking, since the level of CSR in the 3′IgH sequences in LPS/αIgD-dextran–treated splenic B cells is similar to that of CSR in the Sγ2a sequence which has a three-fold higher density of AID target motifs with a much higher percentage of the canonical AGCT motif in the 600-bp core Sγ2a (*SI Appendix*, Fig. S1*G*), indicating that factors beyond AID-targeting motif density (transcription levels, interaction with CSRC, etc.) can influence overall CSR-junction frequency. Thus, these studies indicate that the 3′IgH CBEs prevent the region just downstream from them from becoming transcriptionally activated, synapsing with Sμ in the CSRC and serving as an ectopically induced S region (“eS”) for CSR and aberrant chromosomal deletions.

## Discussion

A prior study reported that deletion of the first eight 3′IgH CBEs had little effect on class switching (17). However, we now demonstrate that complete deletion of all 10 3′IgH CBEs significantly decreases germline transcription and CSR of upstream Sγ3, Sγ2b, Sγ2a, and Sε regions, albeit to varying degrees, after stimulation with αCD40/IL4 or LPS/αIgD-dextran. In addition, class-switching to IgA in 3′IgH CBEs-deleted CH12F3 cells was also reduced to about 50% of control levels (15). Taken together, these findings indicate that the 3′IgH CBEs variably promote CSR to all upstream S regions except Sγ1, likely by focusing 3′IgHRR region transcriptional enhancing activity on the IgH locus, as opposed to being diverted in part to regions downstream of the IgH locus. In the latter context, it is notable that Sγ1 is not affected by the 3′IgH CBE deletion, which is consistent with Sγ1 known to be far less dependent on 3′IgHRR for CSR than other S regions (8, 13, 20, 21). Mechanistically, our findings also show that 3′IgH CBEs deletion creates an eS region just downstream of 3′IgH CBEs for aberrant convergent transcription by the 3′IgHRR enhancer, synapsis with Sμ in the CSRC, and CSR-related deletional joining with the donor Sμ, suggesting that, to some degree, this downstream eS might compete for 3′IgHRR activity with the upstream I promoters in the context of promoting aberrant CSR-related rearrangements in the absence of the 3′IgH CBEs. Overall, our findings implicate the 3′IgH CBEs as insulators that safeguard the integrity of the normal CSR process by isolating the 3'IgHRR (CSRC) activities within the IgH domain and preventing 3′IgHRR off-target activity outside of the IgH domain that leads to aberrant, CSR-mediated chromosomal deletions.

## Materials and Methods

### Generation of Targeted ES Cells and Chimeric Mice.

All 10 3′IgH CBEs were deleted by a standard gene-targeted deletion approach (26) on both alleles of the WT TC1 mouse ES cells, which were derived from a 129/SV mouse. These homozygous 3′IgH CBEs-deleted ES cells were confirmed by PCR genotyping (*SI Appendix*, Fig. S1 *A* and *B*). Subsequently, we deleted both copies of the Aicda (AID) gene from the homozygous 3′IgH CBEs-deleted ES cells via the Cas9/guide RNA (gRNA) approach. The gRNA oligonucleotides for the CRISPR/Cas9 used were cloned into the pX330 vector (Addgene plasmid ID 42230) (27). For CSR-HTGTS-seq experiments, the 3′IgH CBEs knockout ES cells were used to generate chimeric mice with totally ES-cell–derived mature B and T lymphocytes via our RDBC (18). For GRO-seq and 3C-HTGTS experiments, the 3′IgH CBEs and AID double-knockout ES cells were used to generate chimeric mice with RDBC. Mouse work was performed under protocols approved by the Institutional Animal Care and Use Committee at Boston Children’s Hospital.

### Cell Culture.

Primary splenic B cells were isolated by a CD43-negative selection kit from chimeric mice and cultured in medium R15 (RPMI1640, 15% fetal bovine serum, L-glutamate, 1× penicillin and streptomycin). Primary splenic B-cell stimulation was performed with αCD40 (1 μg/mL, eBioscience) plus IL4 (20 ng/mL, PeproTech) or with LPS (25 ng/mL, sigma) plus αIgD-dextran (3 ng/mL) for 96 h. CSR-HTGTS-seq was performed in AID-proficient cells stimulated for 96 h (19). GRO-seq and 3C-HTGTS were performed in AID-deficient cells as previously described (11). Previous studies measured the transcription and interaction of S regions after stimulating the cells for 48 h (10, 28), while the CSR was usually measured after stimulating the cells for longer times (10, 19). To make a better comparison between CSR, S region transcription, and chromatin interactions, we assayed all parameters in splenic B cells stimulated for 96 h.

### Flow Cytometric Analysis.

Flow cytometric analysis was used for measuring IgH class switching in splenic B cells stimulated with either αCD40/IL4 or LPS/αIgD-dextran for 96 h. After 96 h of stimulation, cells were collected and washed once with PBS. Then, the cells were stained for the surface makers with the indicated antibodies (APC-IgG1, APC-IgM/PE-IgG3, and APC-IgM/PE-IgG2b). The APC-IgM and APC-IgG1 antibodies were diluted 100 times from stock concentration, while the PE-IgG3 and PE-IgG2b antibodies were diluted 200 times from stock concentration at room temperature for 10 min. The stained cells were washed once with PBS and resuspended in PBS for flow cytometric analysis with a BDFACSCalibur (BD Bioscience). CellQuest Pro alias software was used for collecting the data, and FlowJo software (10.0.6) was used for analyzing the data.

### CSR-HTGTS-seq and Data Analysis.

CSR-HTGTS-seq libraries generated with a 5′Sμ bait (11, 19) were prepared from primary splenic B cells stimulated with αCD40/IL4 or LPS/αIgD-dextran for 96 h. A total of 25 μg gDNA from αCD40/IL4 or LPS/αIgD-dextran–stimulated splenic B cells was sonicated (25 s ON and 60 s OFF, two cycles with low-energy input) on a Diagenode Bioruptor sonicator. The sonicated DNA fragments were amplified by LAM-PCR with biotinylated 5′Sμ primer. The LAM-PCR products were enriched with streptavidin C1 beads (Thermo Fisher Scientific, #65001) for 4 h at room temperature. The enriched biotin-labeled LAM-PCR products were ligated with adaptor, followed by nested PCR with barcode primers and tag PCR with P5-I5 and P7-I7 primers. The 500- to 1,000-bp tag-PCR products were purified by separation on 1% Tris-acetate-EDTA (TAE) gel. CSR-HTGTS-seq libraries were sequenced by paired-end 150-bp sequencing on a Next-SeqTM550 (Illumina). More details of the method and analysis have been described (11, 19).

Libraries were processed via our published pipeline (29) and mapped against the AJ851868/mm9 hybrid genome as described previously (30). Data were analyzed and plotted after removing duplicates (11, 19). Each experiment was repeated three times for statistical analyses. The junction numbers within different S regions, as well as the percentage analysis of different S-region junctions with respect to total junctions within the C_H_-containing portion of the IgH, are listed in *SI Appendix*, Tables S1 and S2. Primers used for CSR-HTGTS-seq are listed in *SI Appendix*, Table S3.

### 3C-HTGTS.

The 3C-HTGTS analyses (31) were performed on AID^−/−^ mature splenic B cells stimulated with αCD40/IL4 or LPS/αIgD-dextran for 96 h as previously described (11). Ten million cells were collected and cross-linked with 2% formaldehyde for 10 min at room temperature. Then the cross-linked samples were quenched with glycine at a final concentration of 125 mM and lysed in the 3C lysis buffer (50 mM Tris⋅HCl, pH 7.5, 150 mM NaCl, 5 mM ethylenediaminetetraacetic acid (EDTA), 0.5% Nonidet P-40, 1% Triton X-100, protease inhibitors). The nuclei were collected and digested with NlaIII enzyme (NEB, R0125) at 37 °C overnight. The digested nuclei samples were ligated with T4 ligase (Promega, M1801) and incubated overnight at 16 °C. The ligated products were treated with Proteinase K (Roche, #03115852001) at 56 °C overnight for de-cross-linking, and the 3C templates were extracted by phenol/chloroform. The 3C-HTGTS libraries were then sequenced by paired-end 150-bp sequencing on Next-Seq550 (Illumina). More details of the method have been described (11, 31). All the 3C-HTGTS libraries were size-normalized to 370,000 total junctions for comparison. For 3C-HTGTS bait interaction frequency analysis, we counted the number of junctions within the indicated bait-interacting locales for both control and experimental groups. For bar graph presentation, the junction number recovered from control groups was normalized to represent 100%, and relative experimental values are listed as a percentage of control values (*SI Appendix*, Tables S1 and S2). Primers used for 3C-HTGTS are listed in *SI Appendix*, Table S3. Each experiment was repeated three times for statistical analyses.

### GRO-seq Analysis.

GRO-seq libraries were prepared from AID^−/−^ mature splenic B cells stimulated with αCD40/IL4 or LPS/αIgD-dextran for 96 h as described (11). Ten million cells were permeabilized with the fresh-made buffer (10 mM Tris⋅HCl, pH 7.4, 300 mM sucrose, 10 mM KCl, 5 mM MgCl2, 1 mM ethylene glycol tetraacetic acid [EGTA], 0.05% Tween-20, 0.1% Nonidet P-40 substitute, 0.5 mM dithiothreitol [DTT], protease inhibitors, and RNase inhibitor) and resuspended in 100 μL of storage buffer (10 mM Tris⋅HCl, pH 8.0, 25% [V/V] glycerol, 5 mM MgCl_2_, 0.1 mM EDTA, and 5 mM DTT). The nuclear run-on reaction was performed by adding 100 μL of 2X run-on mix (5 mM Tris⋅HCl, pH 8.0, 2.5 mM MgCl2, 0.5 mM DTT, 150 mM KCl, 0.5 mM ATP, 0.5 mM CTP, 0.5 mM GTP, 0.5 mM BrUTP, RNase inhibitor, 1% sarkosyl) at 37 °C for 5 min. RNA was extracted by TRIzol. Hydrolysation was performed by adding NaOH at a final concentration of 0.2 N on ice for 18 min and followed by quenching with ice-cold Tris⋅HCl, pH 6.8, and exchanging buffer via Bio-Rad P30 columns. Then the purified RNA was incubated with BrdU antibody-conjugated beads (Santa Cruz Biotechnology, sc-32323-ac) for 1 h. The enriched run-on samples were incubated with RppH (NEB, M0356S) for 1 h and hydroxyl repair with T4 PNK (NEB, M0201S) for another 1 h. RT-PCR was performed after the 5′ and 3′ RNA adaptor ligation. The complementary DNA template was subjected to making GRO-seq libraries by two rounds of PCR. PCR products of 200 to 500 bp from the first round of PCR were purified by separation on 2.5% TAE gel and subjected to the second round of PCR. The final PCR products were further purified by size-selection with SPRIselect beads (Beckman Coulter, B23318). GRO-seq libraries were sequenced via paired-end 150-bp sequencing on a Next-Seq550 and normalized to a coverage of 10 million 100-nucleotide reads for display. Transcriptional activity of specific regions was calculated as Reads Per Million Reads (RPM) (*SI Appendix*, Tables S1 and S2). Each experiment was repeated three times for statistical analyses.

### Statistical Analysis.

An unpaired two-tailed Student *t* test was used to examine the significant difference between samples. At least three repeats were done for each statistical analysis. Quantitative data are mean ± SD; *n.s.* indicates *P* > 0.05, *P ≤ 0.05, **P ≤ 0.01, ***P ≤ 0.001.

## Supplementary Material

Supplementary File

## Data Availability

All study data are included in the article and supporting information. CSR-HTGTS-seq, GRO-seq, and 3C-HTSTS sequencing raw data analyzed here has been deposited in the Gene Expression Omnibus database under accession number GSE152193.
