# Supplementary material for: Physiological role of the 3′IgH CBEs super-anchor in antibody class switching
Source: Proc Natl Acad Sci U S A. 2021 Jan 13;118(3):e2024392118. doi: 10.1073/pnas.2024392118 (PMC7826415; doi:10.1073/pnas.2024392118)
Supplement: Supplementary File [file pnas.2024392118.sapp.pdf]

PNAS
www.pnas.org

Supplementary Information for

Physiological role of the 3'IgH CBEs super-anchor in antibody class switching

Xuefei Zhang[a,b,c,1], Hye Suk Yoon[a,b,c,2], Aimee M. Chapdelaine-Williams[a,b,c], Nia Kyritsis[a,b,c], Frederick W. Alt[a,b,c,1]

[a]Howard Hughes Medical Institute, Boston Children's Hospital, Boston, MA, 02115

[b]Program in Cellular and Molecular Medicine, Boston Children's Hospital, Boston, MA 02115

[c]Department of Genetics, Harvard Medical School, Boston, MA 02115

[2]Current Address: Regeneron pharmaceuticals, 777 Old Saw Mill River Rd, Tarrytown, NY 10591

[1]To whom correspondence may be addressed. Email: Xuefei.zhang@childrens.harvard.edu or Alt@enders.tch.harvard.edu.

This PDF file includes:

Figures S1 to S5
Tables S1 to S3

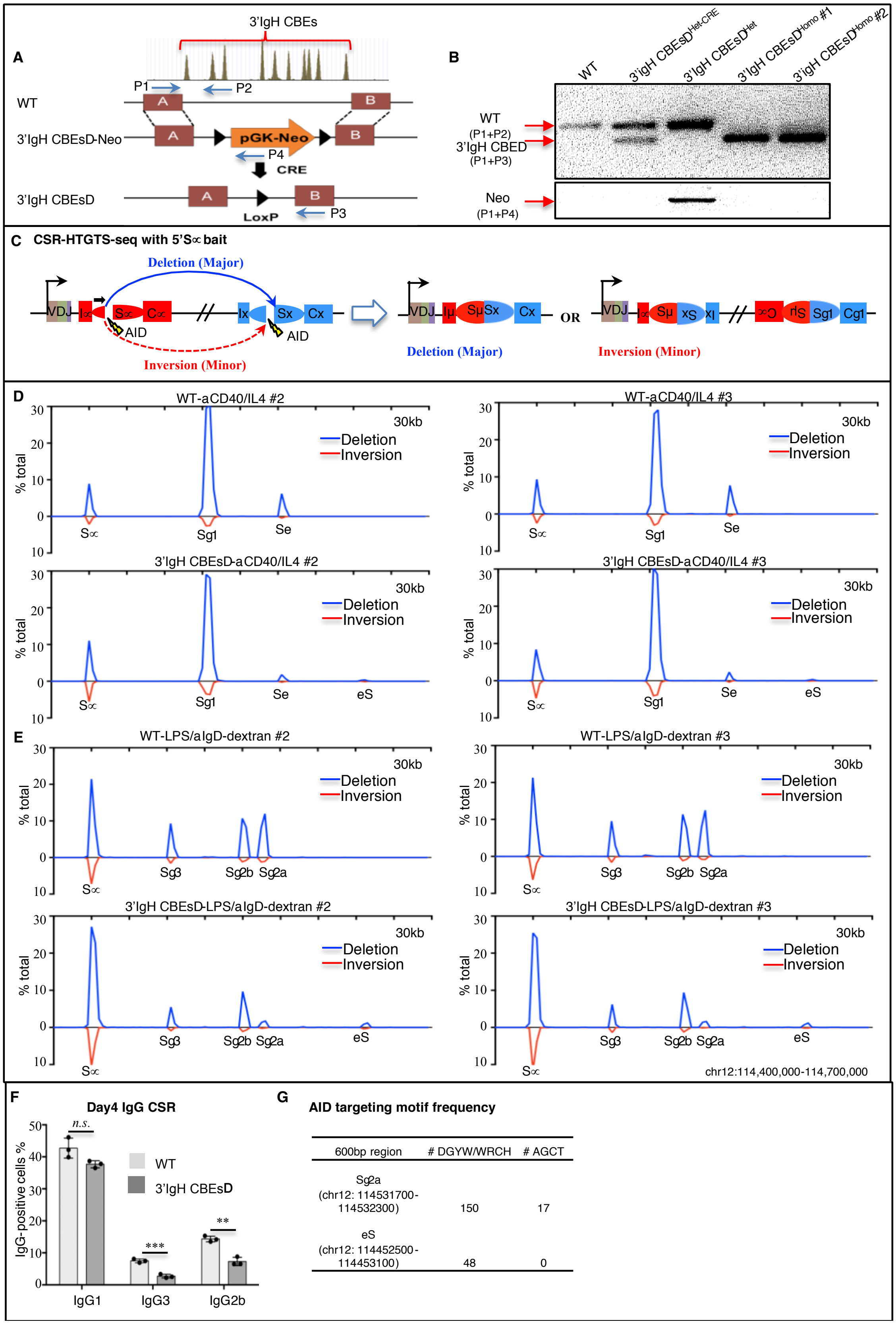

600bp region	# DGYW/WRCH	# AGCT
Sγ2a (chr12: 114531700-114532300)	150	17
eS (chr12: 114452500-114453100)	48	0

Fig. S1. 3'IgH CBEs deletion decreases CSR to most upstream S region and induces aberrant translocation to the eS region.

(A) Illustration of the targeting strategy used to generate the 3'IgH CBEs-deleted TC1 ES cells. Primer1 (P1) and primer2 (P2) were used for amplifying the WT band. Primer1 (P1) and Primer3 (P3) were used for amplifying the 3'IgH CBEs-deleted band. Primer1 (P1) and primer4 (P4) were used for amplifying the part of pGK-neo band. (B) PCR genotyping to confirm the 3'IgH CBEs-deleted ES clones. (C) Illustration of the detection of CSR by CSR-HTGTS-seq with 5'Sμ bait. As indicated, the vast majority of CSR events are deletional, with an upstream end of an Sμ DSB joining to the downstream end of an acceptor S region DSB. (D) Additional two repeats of CSR-HTGTS-seq data shown in Fig. 1B for αCD40/IL4-stimulated WT and 3'IgH CBEs-deleted splenic B cells. The blue lines indicate deletional joining and the red lines indicate inversional joining. (E) Additional two repeats of CSR-HTGTS-seq data shown in Fig. 2A for LPS/αIgD-dextran-stimulated WT and 3'IgH CBEs-deleted splenic B cells. The blue lines indicate deletional joining and the red lines indicate inversional joining. (F) FACS analysis of IgG1 in αCD40/IL4-stimulated splenic B cells, or IgG3 and IgG2b surface expression in LPS/αIgD-dextran-stimulated splenic B cells. Bar graph shows percentages of IgG1, IgG3 and IgG2b expression from the stimulated splenic B cells. Data represents mean ± s.d. from three independent repeats. *P* values were calculated via unpaired two-tailed *t*-test, *n.s.* indicates $p > 0.05$, ** $p \leq 0.01$, *** $p \leq 0.001$. (G) AID-targeting-motif frequency analysis of the 600bp "core" eS and Sγ2a region.

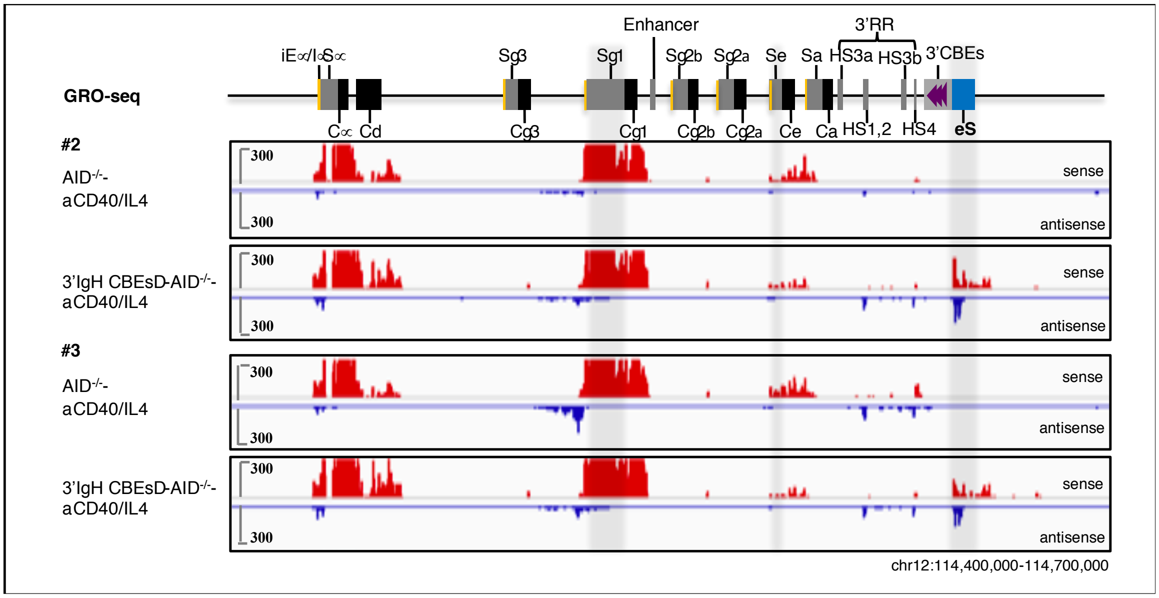

Fig. S2. 3'IgH CBEs deletion decreases Sε transcription after stimulation with αCD40/IL4 and induces transcription across the downstream eS region.

Additional two repeats of GRO-seq data shown in Fig. 3A for αCD40/IL4-stimulated AID-deficient WT and 3'IgH CBEs-deleted splenic B cells. Sense transcription is shown above in red and antisense transcription is shown below in blue lines. Grey bars highlight the Sγ1 and Sε. A blue bar highlight the ectopic S region (labeled as "eS") just downstream of 3'IgH CBEs.

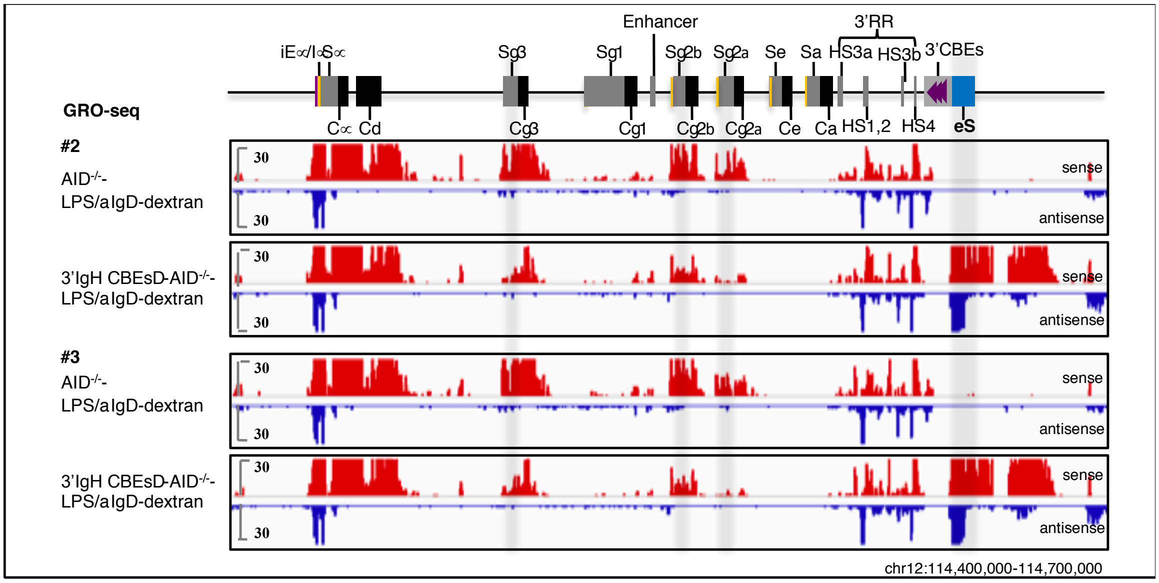

Fig. S3. 3'IgH CBEs deletion decreases Sγ3, Sγ2b and Sγ2a transcription after stimulation with LPS/αIgD-dextran and induces transcription of the eS region.

Additional two repeats of GRO-seq data shown in Fig. 3C for LPS/αIgD-dextran-stimulated AID-deficient WT and 3'IgH CBEs-deleted splenic B cells. Sense transcription is shown above in red and antisense transcription is shown below in blue lines. Grey bars highlight the Sγ3, Sγ2b, Sγ2a, HS3a, HS1,2, HS3b, HS4 and 3'IgH CBEs. A blue bar highlight the ectopic S region (labeled as "eS") just downstream of 3'IgH CBEs.

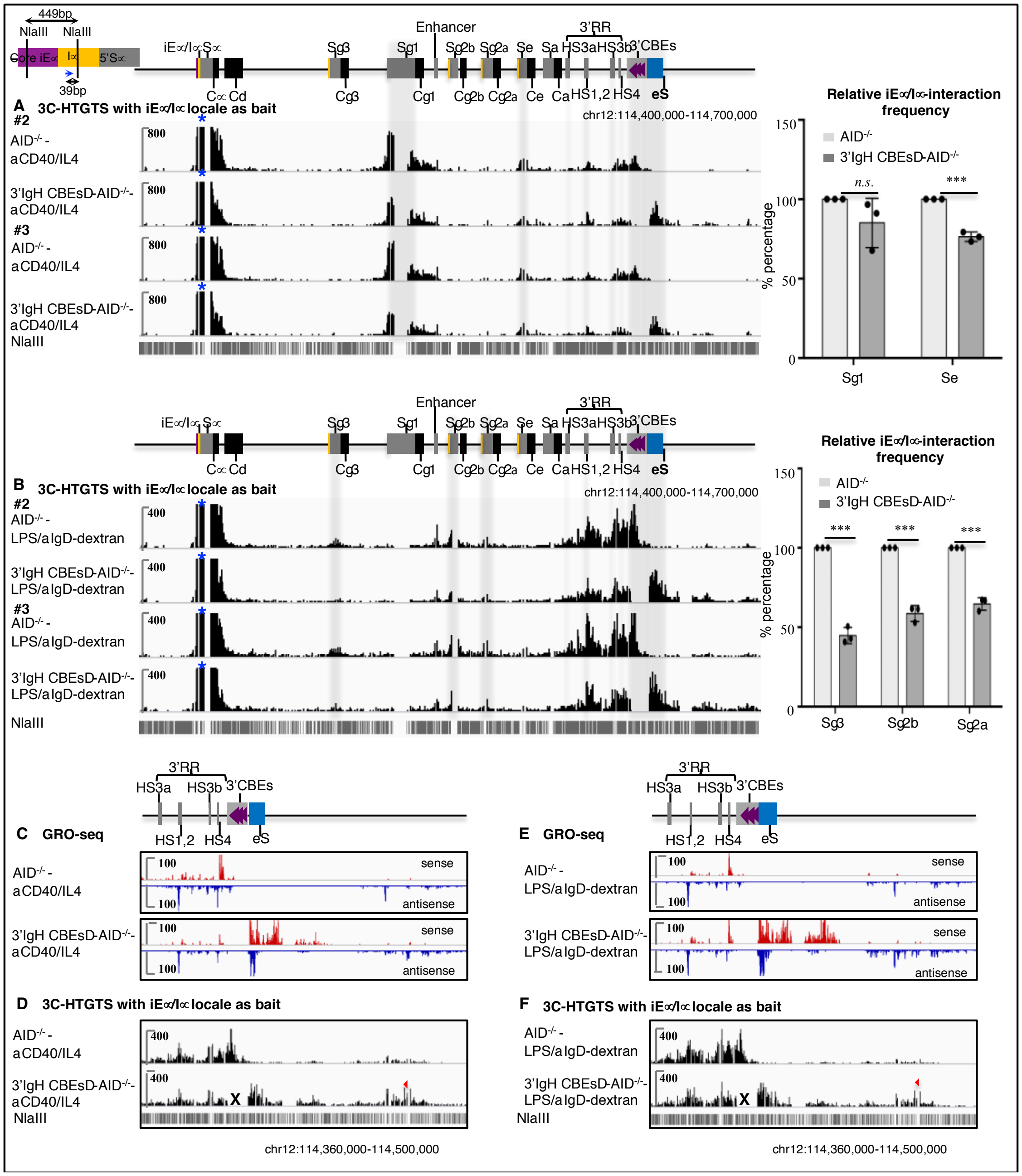

Fig. S4. 3'IgH CBEs deletion decreases most S-S synapsis and induces Sμ-eS synapsis for abnormal rearrangement.

(A) Left: Additional two repeats of 3C-HTGTS data shown in Fig. 4A for αCD40/IL4-stimulated AID-deficient WT and 3'IgH CBEs-deleted splenic B cells. Right: Bar graph shows the relative iEμ-Sμ interaction frequency with Sγ1 and Sε in αCD40/IL4-stimulated splenic B cells. Data represents mean ± s.d. from three

independent repeats. *P* values were calculated via unpaired two-tailed *t*-test, *n.s.* indicates $p > 0.05$, *** $p \leq 0.001$. The raw data for this bar graph is summarized in Table S1. (B) Left: Additional two repeats of 3C-HTGTS data shown in Fig. 4E for LPS/αIgD-dextran-stimulated AID-deficient WT and 3'IgH CBEs-deleted splenic B cells. Right: Bar graph shows the relative iEμ-Sμ interaction frequency with Sγ3, Sγ2b and Sγ2a in LPS/αIgD-dextran-stimulated splenic B cells. Data represents mean ± s.d. from three independent repeats. *P* values were calculated via unpaired two-tailed *t*-test, *** $p \leq 0.001$. The raw data for this bar graph is summarized in Table S2. (C and E) Magnified GRO-seq profiles to better reveal the transcriptional activation of the region downstream of 3'IgH CBEs after 3'IgH CBEs deletion. (D and F) Magnified 3C-HTGTS profiles to better reveal the interaction between iEμ/Iμ bait with the impediments downstream of 3'IgH CBEs after 3'IgH CBEs deletion.

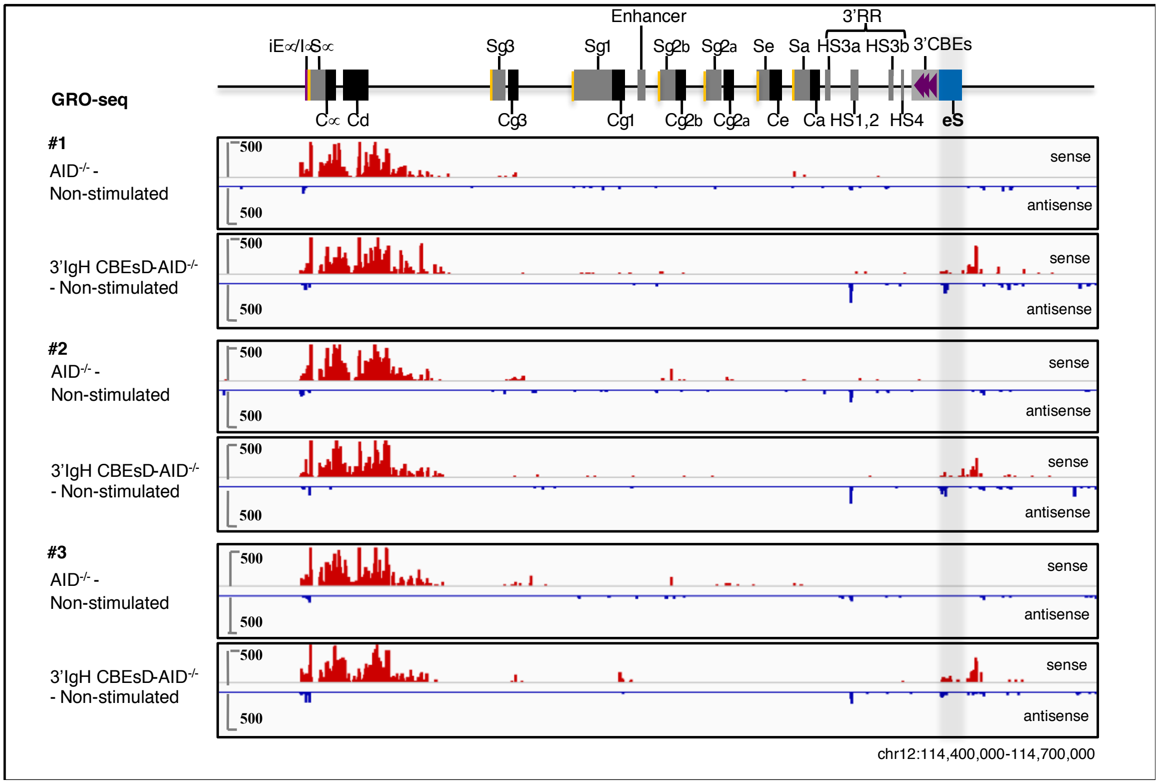

Fig. S5. 3'IgH CBEs deletion induces transcription of the eS region in non-stimulated splenic B cells.

GRO-seq profiles of IgH locus from non-stimulated AID-deficient WT and 3'IgH CBEs-deleted splenic B cells. Sense transcription is shown above in red and antisense transcription is shown below in blue lines. A blue bar highlight the ectopic S region (labeled as "eS") just downstream of 3'IgH CBEs.

Table S1. The effect of 3'IgH CBEs deletion on switching, transcription and interaction of different S regions in WT and 3'IgH CBEsΔ splenic B cells stimulated with αCD40/IL4.

Relative utilization of different S regions in WT and 3'IgH CBEsΔ splenic B cells stimulated with αCD40/IL4.

αCD40/IL4	IgH	Sγ1	Sε	eS
WT #1	9090 (100%)	6722 (73.9%)	966 (10.6%)	0 (0%)
WT #2	11284 (100%)	8660 (76.8%)	1023 (9.1%)	0 (0%)
WT #3	8915 (100%)	6498 (72.9%)	1022 (11.5%)	0 (0%)
3'IgH CBEsΔ #1	5077 (100%)	3785 (74.6%)	185 (3.6%)	35 (0.7%)
3'IgHCBEsΔ #2	6320 (100%)	4767(75.4%)	183 (1.9%)	54 (0.9%)
3'IgH CBEsΔ #3	3750 (100%)	2920 (77.9%)	119 (3.2%)	27 (0.7%)

Relative transcription activity (RPM) of different S regions in AID-deficient WT and 3'IgH CBEsΔ splenic B cells stimulated with αCD40/IL4.

αCD40/IL4	Sγ1	Sε	eS
WT #1	1109	128	0
WT #2	1558	168	0
WT #3	1593	175	0
3'IgH CBEsΔ #1	1184	71	105
3'IgH CBEsΔ #2	1080	65	75
3'IgH CBEsΔ #3	1103	63	76

Relative interaction between iEμ-Sμ locale and different S regions in AID-deficient WT and 3'IgH CBEsΔ splenic B cells stimulated with αCD40/IL4.

αCD40/IL4	Sγ1	Sε	eS
WT #1	100	100	100
WT #2	100	100	100
WT #3	100	100	100
3'IgH CBEsΔ #1	91	79	1226
3'IgH CBEsΔ #2	97	73	1230
3'IgH CBEsΔ #3	68	76	2075

Table S2. The effect of 3’IgH CBEs deletion on switching, transcription and interaction of different S regions in WT and 3’IgH CBEsΔ splenic B cells stimulated with LPS/αIgD-dextran.

Relative utilization of different S regions in WT and 3’IgH CBEsΔ splenic B cells stimulated with αCD40/IL4.

LPS/aIgD-dextran	IgH	Sγ3	Sγ2b	Sγ2a	eS
WT #1	3933 (100%)	564 (14.3%)	857 (21.8%)	902 (22.9%)	1 (0.02%)
WT #2	4940 (100%)	680 (13.7%)	1039 (21.0%)	1138 (23.0%)	0 (0%)
WT #3	6097 (100%)	875 (14.4%)	1285 (21.1%)	1385 (22.7%)	0 (0%)
3’IgH CBEsΔ #1	2087 (100%)	175 (8.4%)	338 (16.2%)	71 (3.4%)	57 (2.7%)
3’IgH CBEsΔ #2	2836 (100%)	216(7.6%)	471 (16.6%)	101 (3.6%)	77 (2.7%)
3’IgH CBEsΔ #3	2958 (100%)	247 (8.4%)	479 (16.2%)	95 (3.2%)	69 (2.4%)

Relative transcription activity (RPM) of different S regions in AID-deficient WT and 3’IgH CBEsΔ splenic B cells stimulated with LPS/αIgD-dextran.

LPS/aIgD-dextran	Sγ3	Sγ2b	Sγ2a	eS
WT #1	69	24	11	0
WT #2	40	16	6	0
WT #3	46	16	6	0
3’IgH CBEsΔ #1	7	5	1	65
3’IgH CBEsΔ #2	7	6	1	61
3’IgH CBEsΔ #3	8	7	1	69

Relative interaction between iEμ-Sμ locale and different S regions in AID-deficient WT and 3’IgH CBEsΔ splenic B cells stimulated with LPS/αIgD-dextran.

LPS/aIgD-dextran	Sγ3	Sγ2b	Sγ2a	eS
WT #1	100	100	100	100
WT #2	100	100	100	100
WT #3	100	100	100	100
3’IgH CBEsΔ #1	41	61	60	1809
3’IgH CBEsΔ #2	51	62	67	1643
3’IgH CBEsΔ #3	43	53	67	2288

Table S3. List of oligos.

Name	Sequences	Purpose
AIDΔ_up_1 (gRNA)	GCCGAAGTCCAGTGAGCAGG	gRNA to delete Aicda gene
AIDΔ_down_1 (gRNA)	GCGAGATGCATTTCGTATGT	gRNA to delete Aicda gene
AIDΔ_up_2 (gRNA)	GTAGGTCTCATGCCGTCCCT	gRNA to delete Aicda gene
AIDΔ_down_2 (gRNA)	GGATTTTGAAAGCAACCTCC	gRNA to delete Aicda gene
5'Sμ_bio	CAGACCTGGGAATGTATGGT	Bio-primer for 5'Sμ bait
5'Sμ_red	CACACAAAGACTCTGGACCTC	Red-primer for 5'Sμ bait
iEμ/Iμ_bio	GGTTATGTAAGAAATTGAAGGACTTT	Bio-primer for iEμ/Iμ bait
iEμ/Iμ_red	TTTCCAATACCCGAAGCATT	Red-primer for iEμ/Iμ bait
